# Optimization of nickel and cobalt biosorption by native *Serratia marcescens* strains isolated from serpentine deposits using response surface methodology

**DOI:** 10.1007/s10661-022-09816-w

**Published:** 2022-02-10

**Authors:** A. Díaz, J. Marrero, G. Cabrera, O. Coto, J. M. Gómez

**Affiliations:** 1 Metal Biotechnology Laboratory, Faculty of Biology, University of Havana (Cuba), Calle 25 #455 Vedado, 10400 La Habana, Cuba; 2 Biological and Enzymatic Reactors Group, Department of Chemical Engineering and Food Technology, Faculty of Sciences, 11510 Puerto Real, Cadiz, Spain

**Keywords:** Biosorption, Nickel, Cobalt, *Serratia marcescens*, Response surface methodology, Serpentine

## Abstract

The treatment of metal-polluted wastes is a challenging issue of environmental concern. Metals can be removed using microbial biomass, and this is an interesting approach towards the design of eco-friendly technologies for liquid waste treatment. The study reported here aimed to optimize nickel and cobalt biosorption from aqueous solutions using three native metal–resistant *Serratia marcescens* strains. Ni(II) and Co(II) biosorption by *S. marcescens* strains was found to fit better to Langmuir’s model, with maximum uptake capacities of 13.5 mg g^−1^ for Ni(II) ions and 19.9 mg g^−1^ for Co(II) ions. Different experimental conditions of initial metal concentration, pH, initial biomass, and temperature were optimized using the Plackett–Burman method, and, finally, biomass and metal concentration were studied using the response surface methodology (RSM) to improve biosorption. The optimum uptake capacities for Co(II) ions by the three biosorbents used were obtained for initial metal concentrations of 35–40 mg L^−1^ and an initial biomass of 6 mg. For Ni(II) ions, the optimum uptake capacity was achieved with 1 mg of initial biomass for *S. marcescens* C-1 and C-19, and with 7 mg for *S. marcescens* C-16, with initial concentrations of 20–50 mg L^−1^. The results obtained demonstrate the viability of native *S. marcescens* strains as biosorbents for Ni(II) and Co(II) removal. This study also contributes to our understanding of the potential uses of serpentine microbial populations for the design of environmental cleanup technologies.

## Introduction

Human activity is one of the main sources of heavy metals in the environment (Volesky, 2007). As chemical or biological processes cannot remove these elements, they accumulate across the trophic network, thus representing a severe threat to life and the ecological balance. For this reason, the treatment of metal-containing wastes has become an issue of environmental and public health concern. One of the most widely used techniques for the remediation of soils contaminated with heavy metals involves reducing mobilization by means of an organic or inorganic sorbent. Indeed, there are several recent publications related to these applications (Tauqeer et al., 2021a, 2021b; Turan, 2021).

Microorganism-based technologies are considered to be feasible alternatives for this purpose, since the physical and chemical treatments of heavy metal–containing effluents is often expensive and generates large amounts of sludge and cannot usually be used to treat metal concentrations below 100 mg L^−1^ (Volesky, 2007).

Biosorption is one of the biotechnological processes that have been proposed for the recovery of heavy metals using biological resources or their derivatives. Biosorption has been studied for many years, and several processes have been patented since 1973 for commercial applications, although biosorption processes have not yet been applied on an industrial scale. This tends to be due to the fact that the mechanism, kinetics, and thermodynamics of the process are not well understood and that the conventional technologies employed to recover metal pollutants based on physical and chemical methods are well established and have been applied successfully. However, conventional physicochemical methods are costly and involve energy-consuming steps while often leading to incomplete metal removal and the generation of waste products. These drawbacks have led to biological approaches being considered as an alternative. Recently, it has been proposed that biosorption-based processes are more suitable for the recovery of precious metals and for waste management to recover metals for resale. This latter advantage is especially important in the mining industry since natural resources on Earth are finite.

Although mining companies have implemented and improved systems for the effective treatment of wastewater, the resulting environmental pollution is still a major concern. Moreover, it is often the case that developing and threshold countries lack treatment facilities in the industrial mining sector since waste management is generally expensive (WDI, 2006).

Biosorption using different microorganisms and their products has been proposed for the design of eco-friendly and cost-effective technologies that could help to minimize the harmful impact of heavy metals in the environment. The main advantages of this approach are its low cost, short operating time, high metal-binding efficiency, improved selectivity for specific metals of interest, reusability of the biosorbent, and the fact that secondary toxic compounds are not produced (Volesky, 2007). Furthermore, this technique is particularly attractive for treating dilute liquid wastes with very low metal concentrations (< 100 mg L^−1^) (Kratochvil & Volesky, 1998).

Metal biosorption has been studied using bacteria (Marrero et al., 2009; Narasimhulu & Setty, 2012; Singh & Gadi, 2012; Gialamouidis et al., 2009), fungi (Chen & Wang, 2007; Cayllahua & Torrem, 2010; Pundir & Dastidar, 2010; Ahmad et al., 2013), algae (Lezcano et al., 2011; Santos et al., 2012; Vijayaraghavan et al., 2005), and several agricultural wastes (Wang & Chen, 2009) as bio-sorbents. Bacteria offer certain advantages due to their small size, adaptability, and growth conditions, which make it relatively easy to obtain them as biosorbents (Wang & Chen, 2009). Additionally, bacteria can be highly effective in uptaking soluble and particulate forms of metals. In this regard, species of *Bacillus*, *Pseudomonas*, *Streptomyces*, *Escherichia*, and *Micrococcus*, among others, have been reported to be good biosorbents for a variety of heavy metals (Kratochvil & Volesky, 1998; Wang & Chen, 2009). Both living and dead bacterial biomasses have been used; although dead biomass has advantages such as a higher affinity for metal ions, it does not require a continuous supply of nutrients and it does not suffer from growth inhibition due to toxic metal concentrations, a problem that usually affects living biomass (Volesky, 2007).

Metal-resistant bacteria have been suggested as potentially efficient biosorbents due to the presence of specific heavy metal binding sites on the cell surface (Kao et al., 2008). For instance, metal-resistant *Escherichia coli* strain WS11 showed a higher Ni(II) and Cd(II) biosorption capacity compared to other non-resistant isolates (Ansari & Malik, 2007). Similarly, *Bacillus thuringiensis* strain OSM29, isolated from metal-contaminated rhizospheric soil, showed tolerance to Cd(II), Cr(II), Cu(II), Pb(II), and Ni(II) ions and its biomass exhibited biosorption capacity for these five metals, with maximum biosorption levels of 59.17, 71.94, 39.84, 30.76, and 43.13 mg g^−1^, respectively (Oves et al., 2013). *Enterobacter cloacae* strain P2B, which is a lead-resistant bacterium, showed the ability to sequester a higher amount of this heavy metal (17% lead by weight) than a non-resistant strain of *E. cloacae* (Naik et al., 2012), and *Enterobacter* sp. J1, isolated from metal-polluted industrial wastewater in Taiwan, was able to tolerate high concentrations of a variety of heavy metals, including lead, copper, cadmium, mercury, zinc, cobalt, and nickel. As such, the latter is considered to be a suitable biosorbent for lead, copper, and cadmium removal (Lu et al., 2006).

Serpentine soils are natural metal-rich ecosystems in which a variety of metal-resistant microorganisms have been characterized (Stoppel & Schlegel, 1995; Mengoni et al., 2001; Park et al., 2004; Pal et al., 2005; Marrero et al., 2007). However, very little research has been carried out on the potential of metal-resistant bacteria from serpentine deposits as suitable biosorbents for heavy metal removal. For example, metal-tolerant *Paenibacillus polymyxa* isolated from serpentine deposits in Turkey showed biosorption capacity for Ni(II) and Cu(II) ions (Rajkumar et al., 2009), and *S. marcescens* strains 16, C-1, and C-4, isolated from serpentine nickel deposits in Moa (Cuba), have a high resistance to Ni(II) and Co(II) and exhibit nickel and cobalt biosorption capacities in monometallic systems (Marrero et al., 2009).

Different processes for the recovery of metals from natural resources or industrial wastes using microorganisms have already been commercialized in applications such as bioleaching (Edelstein, 2011; Schippers et al., 2013), bio-oxidation, and phytomining (van der Enta et al., 2013).

Although biosorption has been extensively studied for many years, the process is still only carried out on a laboratory scale and has yet to be applied on an industrial scale in the mining sector (Fomina & Gadd, 2014). For this reason, the study of biosorption is a high priority in order to optimize the process.

The conventional method used to optimize biosorption is the “one factor at a time” method, in which one independent variable is changed while keeping all others at a set level. This approach may lead to unreliable results and less accurate conclusions, and it also requires many experiments, which may result in the prediction of “false” optimum values (Namdeti & Pulipati, 2013). These drawbacks can be overcome by optimizing all the influential parameters by using an experimental design that allows the study of all factors that influence the biosorption performance and the possible interactions between them. The response surface methodology (RSM) has proven to be a useful tool for studying the interactions of two or more variables that influence the performance of bacterial biosorbents (Lu et al., 2006; Kumar & Singh, 2009; Kiran & Thanasekaran, 2011; El-Ahwany, 2011), to evaluate the interactive effects of operational parameters, to reduce the number of experiments, and to allow researchers to better understand the process under investigation.

The aim of the work reported here was to investigate the Ni(II) and Co(II) biosorption capacity of native metal–resistant *S. marcescens* strains C-1, 16, and 19 and to optimize the parameters initial metal concentration, pH, initial biomass, and temperature using RSM in order to improve biosorption.

## Materials and methods

### Biosorbent preparation

*S. marcescens* strains C-1, C-16, and C-19 were cultivated in Luria–Bertani liquid medium and incubated in a rotary shaker at 150 rev·min^−1^ for 10 h at 37 °C. Cells were harvested by centrifugation at 10,000 rev·min^−1^ for 10 min at 4 °C, and the pellet was washed twice with distilled water. Finally, the cell pellet was dried in an oven at 60 °C for 2 h. The resulting biomass was ground to a smaller particle size using a mortar and pestle, and the sample was stored under dry conditions prior to use.

### Batch biosorption experiments

Biosorption experiments were carried out using 50-mL Erlenmeyer flasks containing 10 mL of metal solution with shaking at 120 rev·min^−1^. After contact, the biomass was harvested by centrifugation at 8000 rev·min^−1^ for 10 min. Supernatants were used to determine the remaining heavy metal concentrations by ICP-AES. The biosorption capacity (*q*) was determined as described previously (Volesky, 2007) according to Eq. (1):

1 $$q(\mathrm{mg\; }{\mathrm{g}}^{-1})=\frac{V\left[L\right]\times \left({C}_{\mathrm{i}}-{C}_{\mathrm{f}}\right)[\mathrm{mg }{\mathrm{L}}^{-1}]}{{X}_{0}[\mathrm{g}]}$$

where *q* is the biosorption capacity, expressed as milligram of metal adsorbed per gram of biomass; *C*_i_ and *C*_f_ are the initial and final metal concentrations, respectively; and *X*_0_ is the initial biomass concentration. Mean values of *q* obtained for each metal were compared by analysis of variance (ANOVA) (*p* < 0.05). Heavy metal solutions were prepared using analytical grade salts of CoSO_4_ × 6H_2_O and NiSO_4_ × 7H_2_O.

To determine the effect of contact time on Ni(II) and Co(II) biosorption by *S. marcescens* strains, experiments were carried out with contact times of 5 min, 15 min, 30 min, 1 h, 2 h, 4 h, and 16 h. Monometallic solutions of each metal were prepared at a concentration of 25 mg L^−1^. The temperature was kept constant at 30 °C, and the pH was maintained at 4.5.

### Sorption isotherms

Experimental data for Ni(II) and Co(II) sorption in monometallic systems were fitted to the mathematical models of Langmuir and Freundlich. Biosorption isotherms were plotted from biosorption tests carried out at different metal concentrations in the range 5–150 mg L^−1^ for Co(II) ions and 5–200 mg L^−1^ for Ni(II) ions.

The Langmuir Eq. (2) provides two parameters: *q*_max_, the maximum metal uptake by the biomass, and the constant *b*, which is inversely proportional to the affinity between metal and biomass (Volesky, 2003). *C*_f_ represents the final metal concentration when sorption equilibrium is reached.

2 $$q={q}_{\mathrm{max}}\frac{b{C}_{\mathrm{f}}}{1+b{C}_{\mathrm{f}}}$$

The nonlinear expression of the Freundlich model, as shown in Eq. (3), also provides two parameters: *K*, a constant related to the biomass sorption capacity, and (1/*n*), which is indicative of the degree of sorption and gives the exponential relationship between the sorption uptake by the biomass (*q*) and the equilibrium metal concentration (*C*_f_) (Volesky, 2003).

3 $$q=K{C}_{\mathrm{f}}^{(\frac{1}{n})}$$

### Effect of different factors on Ni(II) and Co(II) uptake by S. marcescens biomass: experimental design

The effects of different factors such as pH, temperature, initial biomass (*X*_0_), and initial metal concentration [Me]_i_ on the Ni(II) and Co(II) biosorption process by *Serratia marcescens* C-1, 16, and 19 biomass were studied using monometallic systems. Firstly, a screening experimental design was carried out using the Plackettt–Burman method in order to determine whether the influence of each factor was significant or not. The range of each variable was defined on the basis of previous reports for Ni(II) and Co(II) biosorption by *S. marcescens* (Marrero et al., 2009), and each parameter was coded at three levels: high (+ 1), medium (0), and low (− 1). The range and corresponding level for each variable used in the Plackett–Burman design are given in Table 1. A total of 22 experiments were formulated using the MODDE 9.0 software package (MKS Umetrics AB, Sweden), including two replicates for each experiment and six central points (Table 2). The selected response variable was the biosorption capacity, as determined according to Eq. (1).

**Table 1:** Experimental ranges and levels for each independent variable used for the Plackett–Burman design

**Variable levels**	**Variable ranges**				
	**[Co]**_**i**_** (mg L**^**−1**^**)**	**[Ni]**_**i**_** (mg L**^**−1**^**)**	***T* (°C)**	**pH**	***X***_**0**_** (mg)**
+ 1	50	50	40	8	20
0	26	27.5	30	6.25	12.5
− 1	2	5	20	4.5	5

**Table 2:** Matrix obtained when screening the experimental design using the Plackett–Burman method. [Me]_i_, initial metal concentration for Ni(II) or Co(II). − 1, low value; + 1, high value; 0, medium value

**Experiment**	**Variable levels**			
	**[Me]**_**i**_** (mg L**^**−1**^**)**	***T* (°C)**	**pH**	***X***_**0**_** (g)**
1	+1	–1	–1	+1
2	+1	+1	–1	–1
3	+1	+1	+1	–1
4	–1	+1	+1	+1
5	+1	–1	+1	+1
6	–1	+1	–1	+1
7	–1	–1	+1	–1
8	–1	–1	–1	–1
9	0	0	0	0
10	0	0	0	0
11	0	0	0	0
12	+1	–1	–1	+1
13	+1	+1	–1	–1
14	+1	+1	+1	–1
15	–1	+1	+1	+1
16	+1	–1	+1	+1
17	–1	+1	–1	+1
18	–1	–1	+1	–1
19	–1	–1	–1	–1
20	0	0	0	0
21	0	0	0	0
22	0	0	0	0

Having identified the variables that significantly influenced Ni(II) and Co(II) uptake by *S. marcescens* biomass, the optimal conditions for biosorption of each metal were determined by means of D-optimal design and RSM. MODDE 9.0 (MKS Umetrics AB, Sweden) was used for the regression and graphical analysis of the data obtained. Each parameter was coded at specific levels according to the results from the Plackett–Burman screening design. Finally, the model validation was verified experimentally using the theoretical optimum values for each parameter.

## Results and discussion

### Effect of contact time on the Ni(II) and Co(II) biosorption capacity

**Fig. 1:**
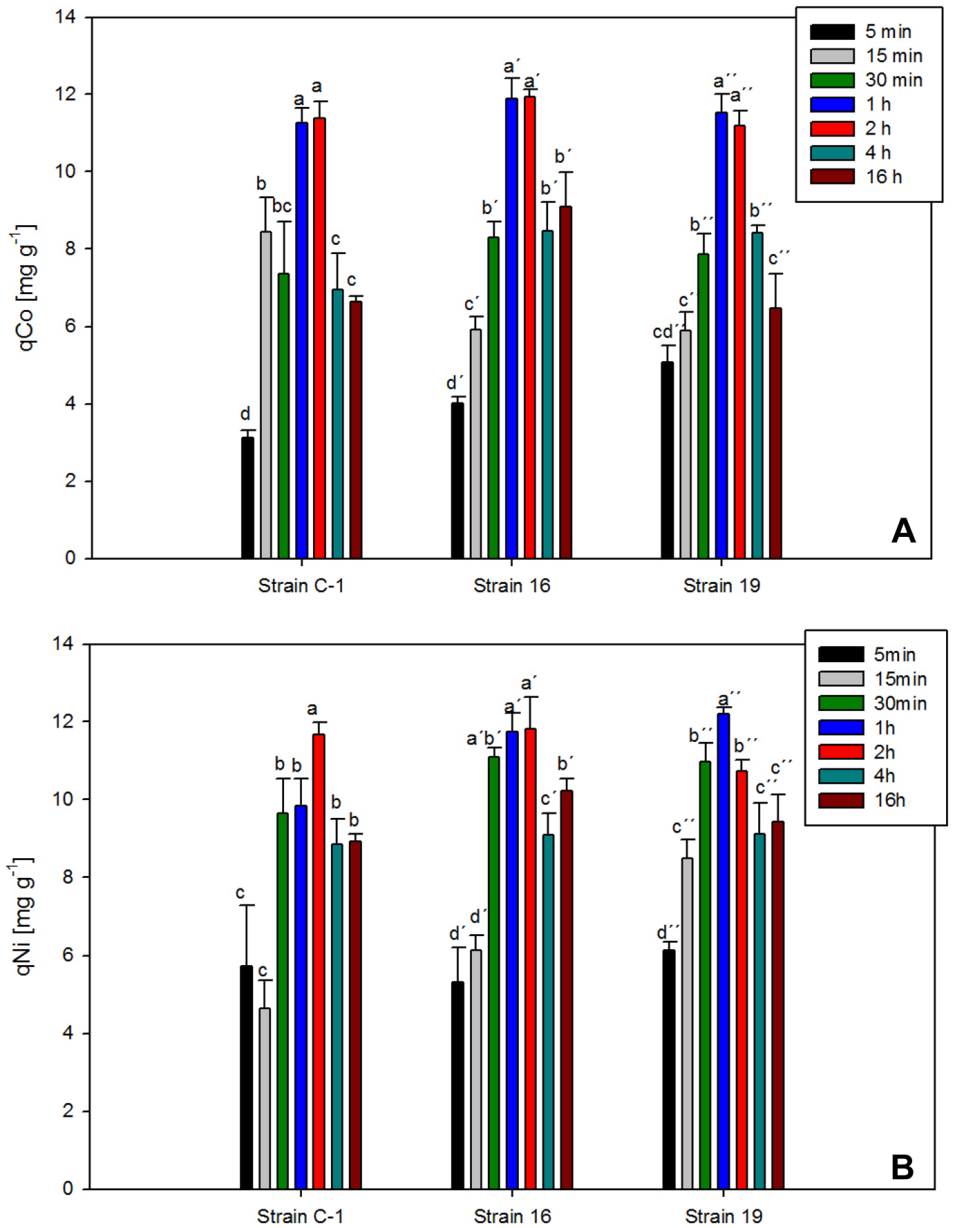
Co(II) and Ni(II) removal capacity by *S. marcescens* strains C-1, 16, and 19, using 25 mg L^−1^ of monometallic solutions of each metal at different contact times. Bars represent the average of three determinations ± *SD*. Different letters express statistically significant differences (*p* < 0.05; *n* = 3)

The biosorption capacity of both metal ions was found to increase from a contact time of 5 min up to 2 h (Fig. 1). The optimal contact time was 1 h for each metal by all strains, and no significant differences in uptake were found between a contact time of 1 h and 2 h, except for Ni(II) by strains C-1 and 19, which showed the highest biosorption capacity at 2 h and 1 h, respectively (Fig. 1).

However, no significant differences were observed in the biosorption capacity of the *S. marcescens* C-1 biomass after 15 min or 30,min of contact with Co(II) solution, as shown by the similar letters obtained after ANOVA of the corresponding *q* values (Fig. 1A). Similarly, *S. marcescens* strains C-1 and 16 did not show differences in Ni(II) biosorption capacity for contact times of 5–15 min and 30–60 min, respectively (see Fig. 1B).

Maximum biosorption values for Co(II) ions were obtained within 1–2 h of contact with this metal solution, with values ranging from 11 to 12 mg g^−1^ for the three strains studied. No significant differences were observed between contact times of 1 and 2 h for the Co(II) solution for all strains studied (Fig. 1A), whereas slight differences were observed between each biosorbent in the presence of Ni(II) ions. Thus, the highest Ni(II) removal capacities displayed by *S. marcescens* C-1 and 16 biomasses were 11.6 and 11.8 mg g^−1^, respectively, for a contact time of 2 h with the metal solution. Strain 16 biomass did not show significant differences for Ni(II) biosorption after contact times of 1 and 2 h, a similar situation to that observed for Co(II) ions. The highest Ni(II) biosorption capacity for strain *S. marcescens* 19 was 12.1 mg g^−1^ for a contact time of 1 h (Fig. 1B).

The Ni(II) and Co(II) biosorption capacity decreased significantly after 2 h for all the biosorbents studied, thus indicating that this was sufficient time for the system to reach equilibrium. This result is consistent with others reported in the literature, where the contact time to reach equilibrium in metal biosorption using bacterial biosorbents has been set at 2 h (Singh & Gadi, 2012; Gialamouidis et al., 2009). Considering these results, a contact time of 2 h between the biomass of *S. marcescens* strains C-1, 16, and 19 and the metal solutions was selected to conduct the subsequent set of experiments.

The lower *q* values obtained for each metal biosorption by *S. marcescens* strains C-1, 16, and 19 at a contact time of 5 min could be due to a lack of sufficient time for the metal-biomass interactions to take place. A reduction in *q* values was also observed for contact times of 4 h and 16 h, although in these cases, a desorption phenomenon possibly takes place once the system has reached the equilibrium state after 1–2 h of metal-biomass contact.

### Sorption isotherms in monometallic systems

**Fig. 2:**
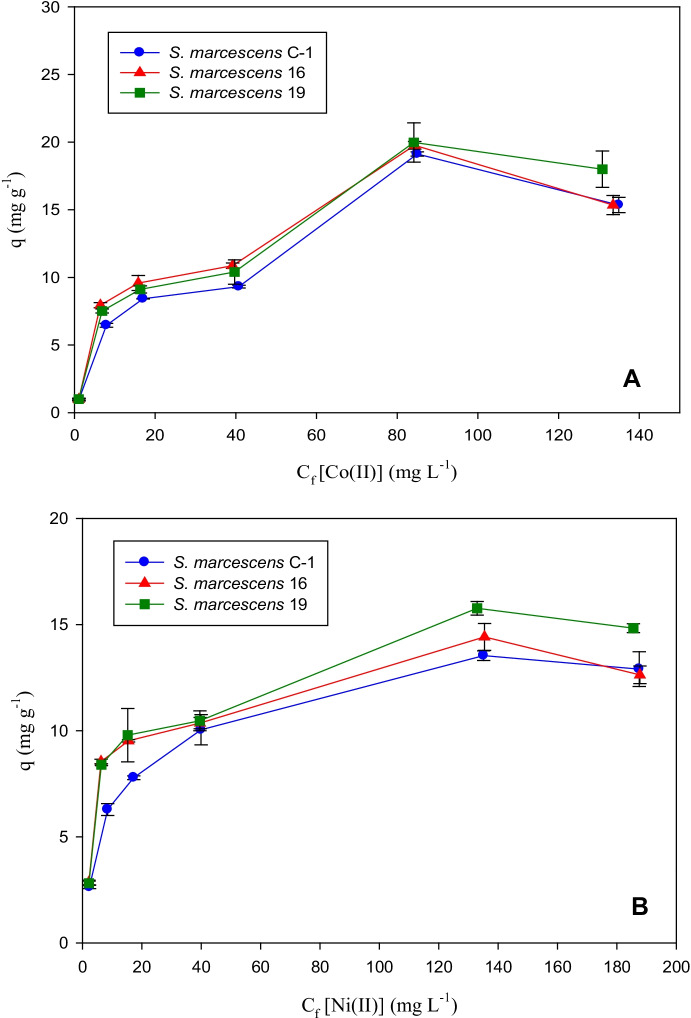
Adsorption isotherms for Co(II) (**A**) and Ni(II) (**B**) ions using *S. marcescens* C-1, 16, and 19 biomass at an initial pH of 4.5 and temperature of 30 °C. Each point represents the average of three determinations ± *SD*

The Ni(II) and Co(II) biosorption isotherms for *S. marcescens* strains C-1, 16, and 19 are shown in Fig. 2. In all cases, an increase in the initial metal concentrations led to an increase in the metal removal capacity up to a certain point, after which further increases in metal concentrations did not produce higher biosorption values.

The highest Co(II) uptake for the three biosorbents studied was obtained for an initial metal concentration of 100 mg L^−1^, with very similar values of 19.1, 19.8, and 20 mg g^−1^ being obtained for *S. marcescens* strains C-1, 16 and 19, respectively. An increase in the biosorption capacity of the biomass was observed in the presence of Ni(II) ions up to an initial metal concentration of 150 mg L^−1^, after which further increases in metal biosorption capacity were not observed on increasing the metal concentration. The maximum *q* values obtained for the biomass of *Serratia marcescens* strains C-1, 16, and 19 were different, with values of 13.5, 14.4, and 15.8 mg g^−1^, respectively.

The behavior observed as regards the biosorption capacity of *S. marcescens* strains 16 and 19 was very similar for both metal cations studied for initial concentrations in the range 5 to 50 mg L^−1^ (Fig. 2). In these cases, the *q* values obtained were higher than those for biomass from *S. marcescens* strain C-1. When the initial concentration of Ni(II) was 50 mg L^−1^, the *q* values were very similar (~ 10 mg g^−1^) for the biomasses from all three strains (Fig. 2B). At initial concentrations greater than 100 mg L^−1^ for Co(II) ions and 50 mg L^−1^ for Ni(II) ions, the highest *q* values were obtained for *S. marcescens* strain 19, with maximum uptake values of 20 mg g^−1^ for Co(II) and 15.8 mg g^−1^ for Ni(II) (Fig. 2).

Experimental data obtained from the equilibrium were fitted to the two mathematical models usually employed in the literature, namely the Langmuir and Freundlich models. The values of the relative constants for both models and the linear regression coefficient (*R*^2^) in each case are given in Table 3. The values of these coefficients indicate a better fit of the experimental data to the Langmuir than to the Freundlich model for the three biosorbents studied in the presence of both metal solutions. On the basis of these results, it can be concluded that the Ni(II) and Co(II) biosorption processes in monometallic solutions by the biomasses of *S. marcescens* strains C-1, 16, and 19 take place in a monolayer, are homogenous with respect to the type and affinity for the metal of the active sites, and the biomass becomes saturated when a large amount of metal is present (Volesky, 2003; Puranik & Paknikar, 1997; Saeed et al., 2005). These findings are consistent with those obtained for several bacterial biosorbents in the presence of monometallic solutions, which usually fit very well to the Langmuir model (Lesmana et al., 2009).

**Table 3:** Langmuir and Freundlich constants obtained for Co(II) and Ni(II) biosorption by *S. marcescens* C-1, 16, and 19 biomasses

**Biomass (strain)**	**Langmuir model**			**Freundlich model**		
	***q***_**max**_** (mg g**^**−1**^**)**	***b***	***R***^**2**^	***K* (mg g**^**−1**^**)**	**1/*n***	***R***^**2**^
**Co(II)**						
*S. marcescens* C–1	18.48	0.062	0.95	1.25	0.58	0.88
*S. marcescens* 16	17.97	0.089	0.95	1.54	0.55	0.80
*S. marcescens* 19	21.33	0.058	0.96	1.44	0.58	0.87
**Ni(II)**						
*S. marcescens* C–1	13.74	0.071	0.97	2.55	0.33	0.88
*S. marcescens* 16	13.25	0.13	0.99	3.56	0.27	0.70
*S. marcescens* 19	15.98	0.093	0.99	3.22	0.32	0.80

The values obtained for the constant *q*_max_ for each metal reveal a higher sorption capacity for Co(II) than for Ni(II) ions. Furthermore, the metal ion affinity of each biosorbent, as deduced from the values of the constant *b* from the Langmuir model (inversely proportional to metal affinity), was found to be higher for Co(II) than for Ni(II) ions (Table 3).

It has been reported in the literature that metal biosorption processes are influenced by both factors such as pH, temperature, and metal and biomass concentrations, among others, that affect the biosorption process itself, as well as by specific characteristics of the metal cations, such as ionic radius and covalent index (directly dependent on electronegativity; Chen & Wang, 2007). The metals used in the present study are both divalent cations, and they have very similar ionic radii. However, the covalent index for Co(II) ions is higher than that for Ni(II) ions (Chen & Wang, 2007), which could explain the higher affinity and biosorption capacity for this metal.

In general, Ni(II) biosorption values reported in the literature are diverse and range from 2 to 100 mg g^−1^. In most cases, values corresponding to *Enterobacteriaceae* are no higher than 15 mg g^−1^ when using initial metal concentrations of 50 mg L^−1^ or lower (Ansari & Malik, 2007; Churchill et al., 1995). For this reason, the results obtained for *Serratia marcescens* strains C-1, 16, and 19, with *q*_max_ values of 13.74, 13.25, and 15.98 mg g^−1^, respectively, are comparable to, and in some cases higher than, those reported in the literature.

Co(II) biosorption studies involving microbial biosorbents are scarce, particularly in the case of bacterial biosorbents (Tripathi & Srivastava, 2007; Abdel-Razek et al., 2009). The study reported here therefore represents a contribution to our knowledge of Co(II) biosorption by bacterial biosorbents, especially with *Enterobacteriaceae*.

### Effect of different factors on Ni(II) and Co(II) uptake by S. marcescens strains C-1, 16, and 19 biomass: experimental designs

#### Screening using the Plackett–Burman method

**Fig. 3:**
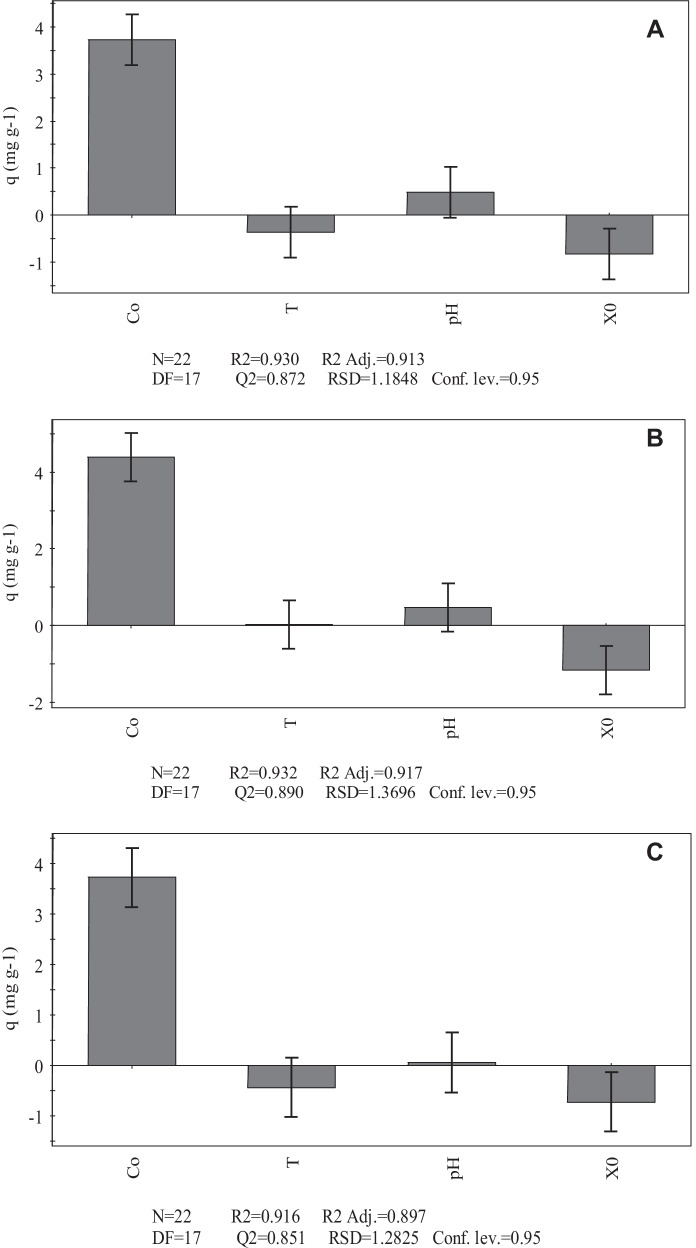
Scaled and centered coefficients for the response variable (*q*) obtained from the Plackett–Burman screening design for Co(II) biosorption by *S. marcescens* strains C-1 (**A**), 16 (**B**), and 19 (**C**)

**Fig. 4:**
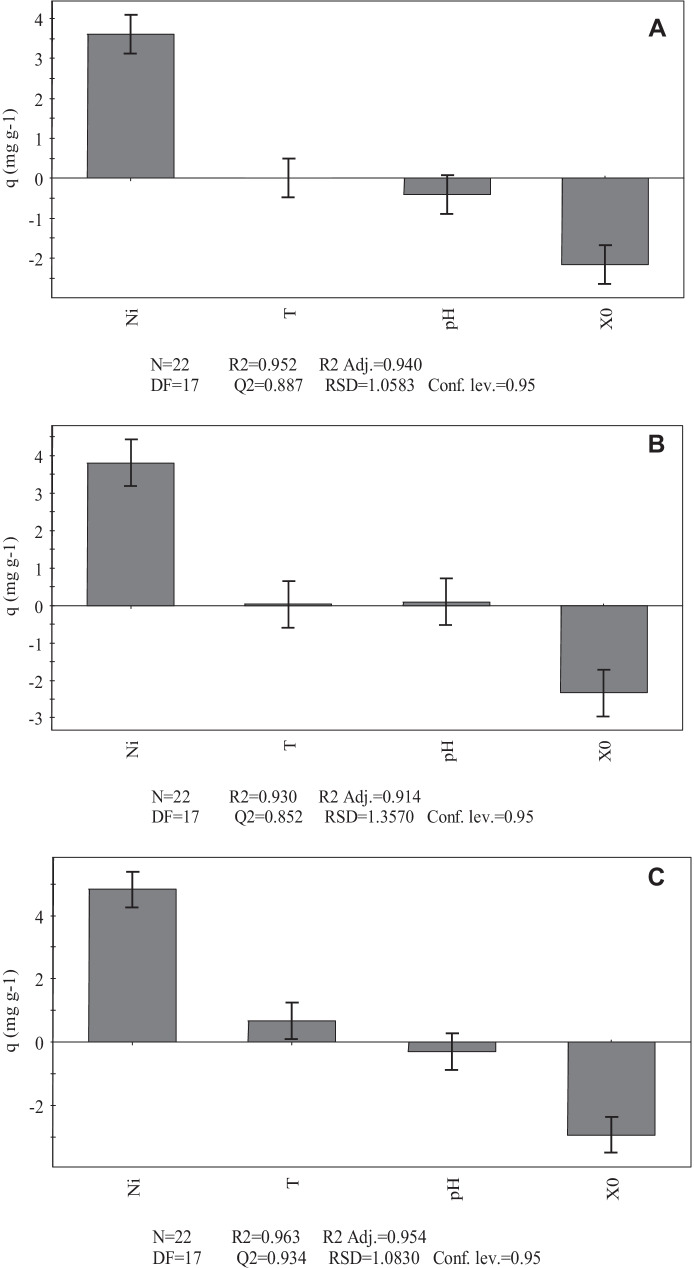
Scaled and centered coefficients for the response variable (*q*) obtained from the Plackett–Burman screening design for Ni(II) biosorption by *S. marcescens* strains C-1 (**A**), 16 (**B**), and 19 (**C**)

The data obtained from screening using the Plackett–Burman method were analyzed using the multiple linear regression (MLR) model to define the influence of each factor studied on the response of each variable. The results obtained show that, for the three biosorbents studied, the initial metal concentration ([Me]_i_) and initial biomass (*X*_0_) have a significant influence on the Ni(II) and Co(II) biosorption capacity (Figs. 3 and 4). The initial metal concentration has a positive influence on the biosorption process, whereas the influence of initial biomass is negative. This means that an increase in initial metal concentration within the studied range leads to an increase in metal removal capacity, while an increase in initial biomass leads to a decrease in biosorption capacity. This latter finding can be explained by taking into account that higher values of initial biomass could lead to aggregation of biosorbent particles, which in turn would limit the number of sites available for metal adsorption.

Similar observations were reported by Gialamouidis et al. (2009), who found that the Ni(II) biosorption capacities of *Pseudomonas* sp. and *Staphylococcus xylosus* biomass decreased with increasing initial biomass concentration. This behavior is analogous to that observed by El-Ahwany (2011) for the Cu(II) biosorption efficiency of the biomass from the lactic acid bacterium *Oenococcus oeni* PSU-1. Studies by Singh and Gadi (2012) with *P. oleovorans* biomass showed a higher biosorption capacity when the initial biomass was increased from 0.4 to 2.4 mg mL^−1^, although further increases in the amount of biomass did not lead to higher metal biosorption.

The influence of pH and temperature within the ranges selected in the present study on the Ni(II) and Co(II) removal capacity was not significant at a confidence level of 95%. This finding was not unexpected because the values selected are usually reported to have no influence on the biosorption performance of microbial biosorbents (Volesky, 2007; Singh & Gadi, 2012; Naja et al., 2009). In particular, the pH values used in the present study were selected according to previous findings from Marrero et al. (2009), who demonstrated that metal biosorption did not occur at a pH of 2 when using biomass from *S. marcescens* strains that were also native to Moa mines. On the other hand, pH values above 8 were not considered in the present study because it has been established by different authors that, under these conditions, Ni(II) and Co(II) hydroxides precipitate, thus leading to an overestimation of the biosorption capacity of the biosorbents (Gialamouidis et al., 2009; Singh & Gadi, 2012).

It can be concluded from the results obtained in the screening design that [Me]_i_ and *X*_0_ are the factors that influence the Ni(II) and Co(II) biosorption process within the range studied. As a result, these parameters were selected for the subsequent experiments.

#### D-optimal design and response surface methodology

A D-optimal design was applied to evaluate the interactions between the two selected factors on the biosorption process by *S. marcescens* biomass. A total of 28 runs were formulated using the MODDE 9.0 software package (MKS Umetrics AB, Sweden), including two replicates for each experiment and two central points. Initial metal concentration ([Me]_i_) was defined as in the Plackett–Burman design, but the initial biomass (*X*_0_) was re-defined as a four-level variable with values of 0.001, 0.0025, 0.005, and 0.01 g, coded as − 1, − 0.111, − 0.667, and 1, respectively, in the model matrix (Tables 4 and 5). There were some differences in the experimental and predicted values of the response variable, as confirmed by the *R*^2^ values obtained, which ranged between 72 and 80% for Co(II) ions and between 70 and 94% for Ni(II) ions.

**Table 4:** D-optimal design matrix in coded terms, with experimental and predicted values for Co(II) sorption capacity (mg g^−1^) by biomass from *S. marcescens* strains

**Runs**	**Factor levels**		**Biosorption capacity** (***q*) (mg g**^−1^**)**					
			***S. marcescens* C-1**		***S. marcescens* 16**		***S. marcescens* 19**	
	**[Co]**	***X***_**0**_	**Exp**	**Pred**	**Exp**	**Pred**	**Exp**	**Pred**
1	–1	–1	7.2733	7.02	7.4333	7.56	7.19333	8.07
2	1	–1	2.9666	5.53	0.3666	4.09	4.26667	4.25
3	0	–1	11.6	11.45	11.1	10.2844	11.1	11.952
4	–1	–0.667	3.9493	3.27	4.1133	5.5593	4.16533	5.5332
5	1	–0.667	9.9066	9.0074	8.4266	8.4911	9.46667	8.6993
6	–1	–0.111	2.2258	2.54	2.3998	3.074	1.88267	2.61
7	1	–0.111	15.5333	12.21	13.093	12.77	12.4333	12.91
8	0	–0.111	9.5	10.515	12.86	12.3773	9.94	11.0054
9	–1	1	1.0503	0.4358	1.1691	0.0252	0.939333	0.2154
10	–1	1	0.8393	0.4358	0.9883	0.0252	1.06333	0.2154
11	1	1	9.2866	10.1962	10.5467	11.1556	10.9167	10.8659
12	1	1	9.7666	10.1962	10.9467	11.1556	10.7767	10.8659
13	0	1	8.44	8.44941	9.98	10.0467	9.41	8.7831
14	0	1	8.78	8.44941	8.95	10.0467	8.89	8.7831
15	–1	–1	7.0533	7.026	7.1133	7.56	8.94333	8.07
16	1	–1	6.4666	7.73798	6.7666	6.4742	3.56667	4.25
17	0	–1	8.5	9.4162	12.1	12.49	9.3	9.4082
18	–1	–0.667	4.1453	4.95548	4.0173	5.5593	4.17733	5.5332
19	1	–0.667	9.5466	9.00744	9.6266	8.4911	8.94667	8.6993
20	–1	–0.111	2.3982	2.54	2.3708	3.074	1.95467	2.61
21	1	–0.111	11.8733	10.359	14.913	12.76	18.0733	12.91
22	0	–0.111	10.32	10.515	11.88	12.3773	9.74	11.0054
23	–1	1	1.1495	0.4358	1.0553	0.0252	1.01233	0.2154
24	–1	1	0.8673	0.4358	1.1010	0.02529	1.1240	0.2154
25	1	1	9.2266	10.1962	10.8267	11.1556	9.2266	10.865
26	1	1	9.2866	10.1962	10.5467	11.1556	8.3766	10.865
27	0	1	8.4	8.44941	9.82	10.0467	9.04	8.7831
28	0	1	8.42	8.44941	8.82	10.0467	8.54	8.7831

**Table 5:** D–optimal design matrix in coded terms, with experimental and predicted values for Ni(II) sorption capacity (mg g^−1^) by biomass from *S. marcescens* strains

**Runs**	**Factor levels**		**Biosorption capacity** (***q***)** (mg g**^−1^**)**					
			***S. marcescens* C–1**		***S. marcescens* 16**		***S. marcescens* 19**	
	**[Ni]**	***X***_**0**_	**Exp**	**Pred**	**Exp**	**Pred**	**Exp**	**Pred**
1	–1	–1	7.0133	7.45	9.42333	8.72	11.2933	10.95
2	1	–1	9.3666	9.73185	3.76667	3.92	5.5666	7.08
3	0	–1	12.5	12.1017	3.5	5.07	11.1	11.0185
4	–1	–0.667	7.8613	7.18028	8.11733	7.65433	7.51333	7.67
5	1	–0.667	11.0667	10.26	11.2667	10.72	10.9867	9.90
6	–1	–0.111	4.9486	4.7	4.9146	6.13	5.6146	5.69
7	1	–0.111	8.4533	8.8	13.113	13.78	11.0733	10.81
8	0	–0.111	10.26	10.56	11.48	10.8659	10.18	10.2494
9	–1	1	2.7183	2.4316	2.9653	2.8455	2.7433	2.4389
10	–1	1	2.6513	2.4316	2.7523	2.8455	2.9303	2.4389
11	1	1	10.5667	9.8985	9.9266	11.4233	9.9266	10.8222
12	1	1	10.3367	9.8985	10.406	11.4233	10.7267	10.8222
13	0	1	9.41	9.2441	8.97	8.0387	8.52	8.6260
14	0	1	8.67	9.2441	9.65	8.0387	8.2	8.6260
15	–1	–1	8.7733	8.3133	9.2233	8.72	11.0833	10.95
16	1	–1	9.2666	9.7318	5.9666	6.2415	7.1666	7.08
17	0	–1	11.8	12.101	4.6	5.07	11.3	11.0185
18	–1	–0.667	7.7213	7.1802	7.9813	7.6543	7.4213	8.6468
19	1	–0.667	9.9466	9.6068	12.1067	10.72	10.4267	9.90
20	–1	–0.111	4.8886	5.4276	4.8126	6.13	5.8046	5.69
21	1	–0.111	8.7133	9.5342	12.4933	11.8228	10.0133	9.7465
22	0	–0.111	12.08	10.56	11.24	10.8659	10.16	10.2494
23	–1	1	2.4383	2.4316	2.6713	2.8455	2.7753	2.4389
24	–1	1	2.5543	2.4316	2.8363	2.8455	2.7723	2.4389
25	1	1	10.3867	9.8985	10.0767	11.4233	10.7267	10.8222
26	1	1	9.23667	9.8985	10.5967	11.4233	10.7467	10.8222
27	0	1	9.37	9.2441	9.44	9.77	8.65	8.6260
28	0	1	8.21	9.2441	9.46	9.77	8.93	8.6260

**Fig. 5:**
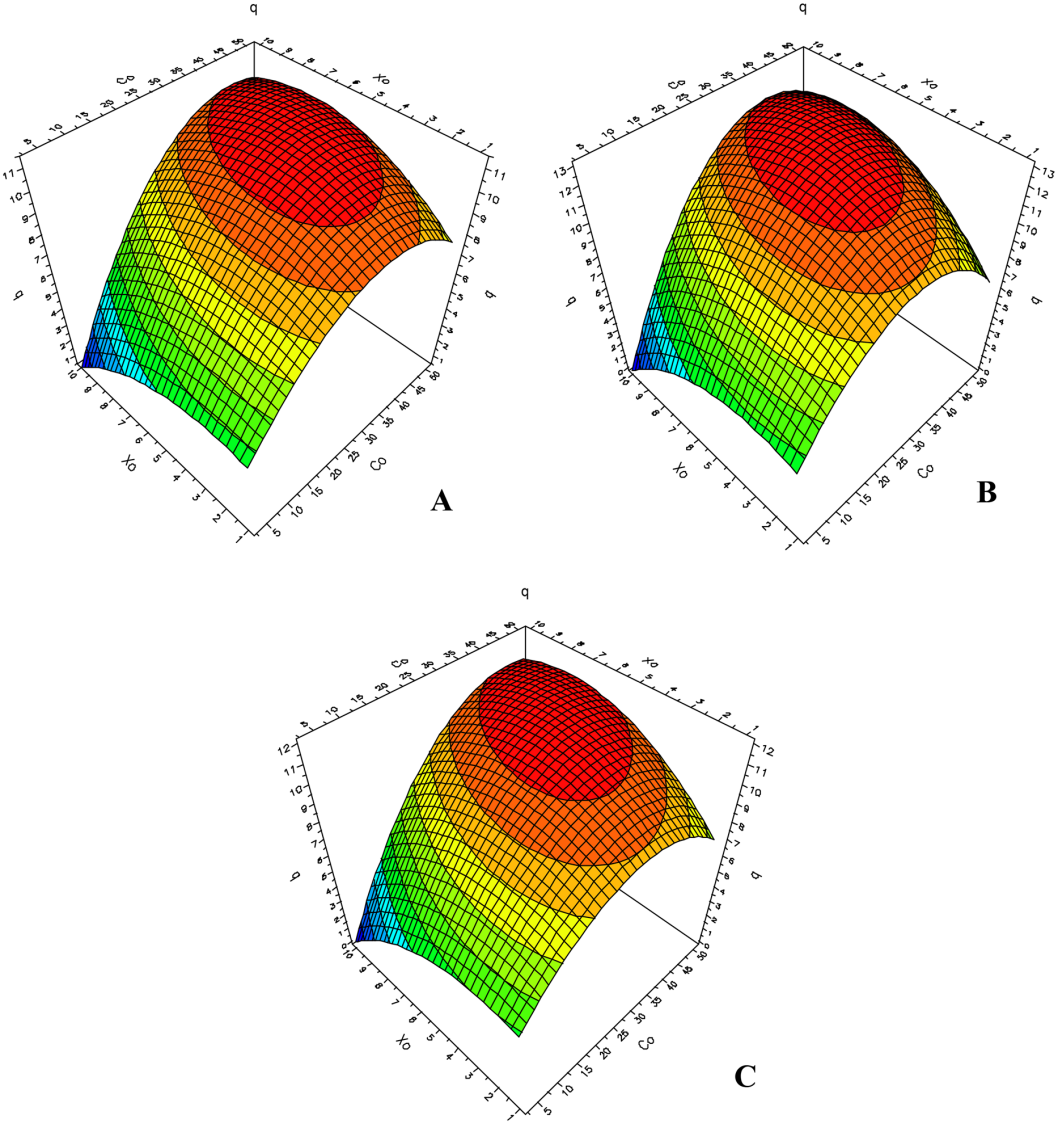
D-optimal contour plots for the combined effect of independent factors on the Co(II) biosorption capacity of *S. marcescens* strains C-1 (**A**), 16 (**B**), and 19 (**C**)

**Fig. 6:**
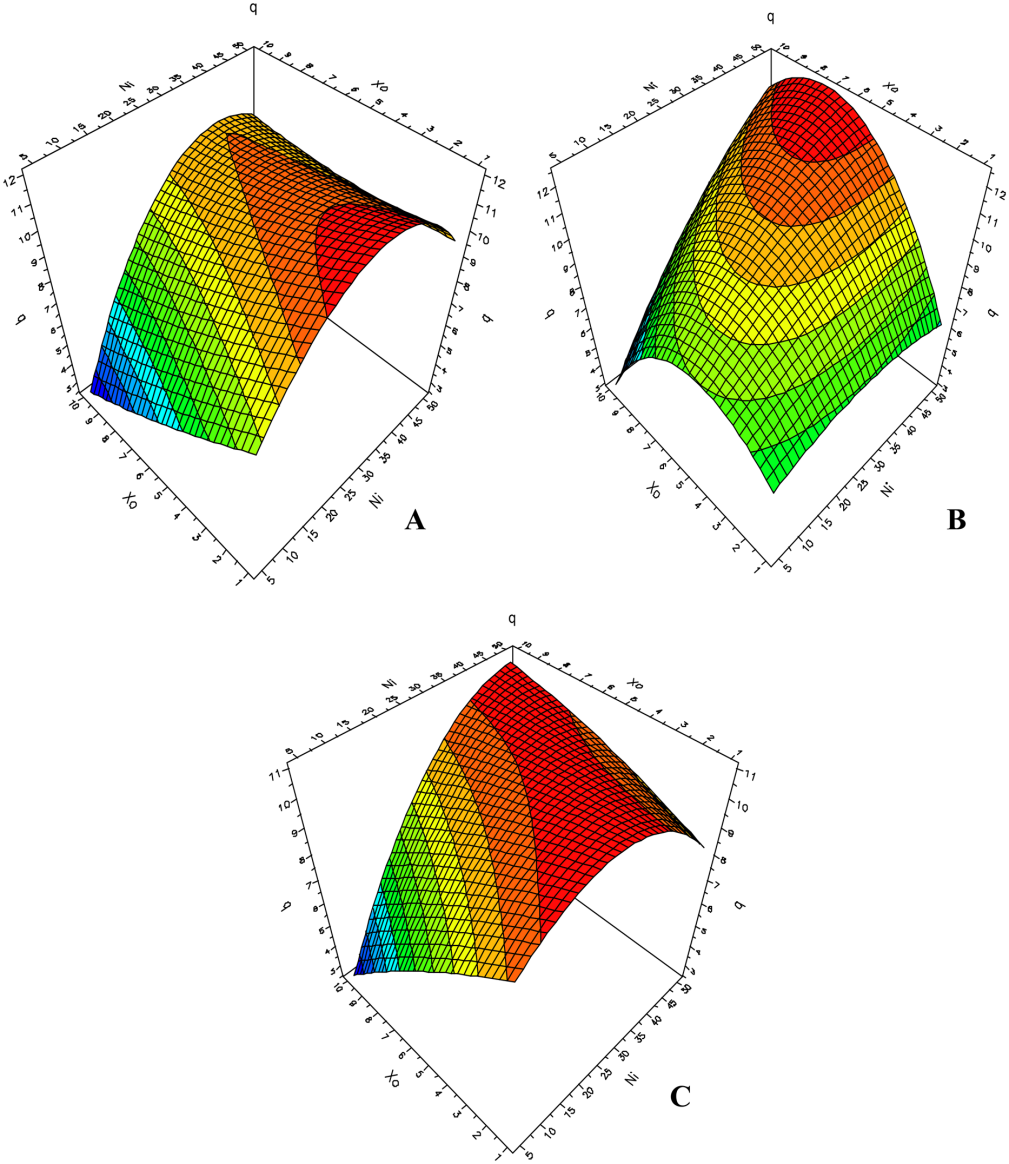
D-optimal contour plots for the combined effect of independent factors on the Ni(II) biosorption capacity of *S. marcescens* strains C-1 (**A**), 16 (**B**), and 19 (**C**)

The 3-D plot for Co(II) and Ni(II) biosorption capacity considering the combined effect that the two factors in question have on the equilibrium metal uptake is shown in Figs. 5 and 6.

The behavior observed for Co(II) biosorption capacity was very similar for all three biosorbents studied (Fig. 5). As can be observed, the Co(II) biosorption capacity increases as the initial metal concentration and biomass values rise until an optimum value is reached, after which further increases in either of these two parameters do not lead to an increase in biosorption capacity. There is a region on the surface response graph where optimum values for the response variables can be achieved. This region is in the concentration range 30–50 mg L^−1^, with an initial biomass in the range 2–9 mg. The *q* values decrease in the zones corresponding to higher values of either biomass or initial metal concentration, a finding that possibly indicates the occurrence of overlapping phenomena that take place when a higher number of biosorbent particles are present. On the other hand, the lower *q* values obtained for low initial biomass values and higher initial metal concentrations could be related to the saturation of metal binding sites on the biosorbent due to the presence of high metal concentrations in conjunction with low biomass.

A different type of behavior was observed for each of the three biosorbents under investigation in the presence of Ni(II) ions (Fig. 6). Thus, for the biomass from strain C-1 (Fig. 6A), optimum values for the response variable were obtained in the presence of initial metal concentrations in the range 25–45 mg L^−1^ and from 0.001 to 0.004 g for the initial biomass. This means that the lower the quantity of biomass in contact with the metal solution, the higher the biosorption capacity of the sorbent. In fact, this result reveals one of the characteristics that make bacterial biosorbents suitable for applications in bioremediation technologies, namely the high surface/volume ratio due to the small size of the particles. This characteristic is responsible for a higher number of exposed active binding sites that can contact with the sorbate (Singh & Gadi, 2012; Vieira & Volesky, 2000). On increasing the initial metal concentration in the presence of these low quantities of biomass, a decrease in the biosorption capacity is observed due to saturation of the binding sites on the biosorbent. As a consequence, initial metal concentrations above 45 mg L^−1^ lead to a reduction in *q* values for this metal.

A different behavior was observed for the biomass from strain 16, for which higher biosorption values were obtained for initial metal concentrations of 40–50 mg L^−1^ and initial biomasses of 5–9 mg (Fig. 6B). In this case, the decrease in *q* values for initial biomasses above 9 mg could be due to overlapping phenomena, whereas saturation by the metal concentration present could be observed with an initial biomass of less than 3 mg.

The behavior of strain 19 proved to be unusual since maximum *q* values were achieved upon using all of the initial biomass values in the range studied, with different initial metal concentrations in each case (Fig. 6C). For example, according to this model, maximum *q* values were obtained when 10 mg of biomass and an initial metal concentration of 50 mg L^−1^ were used. Furthermore, maximum Ni(II) biosorption values were achieved when using initial metal concentrations of 20–40 mg L^−1^ and the lowest biomass values.

The main objective of this methodology is to determine the optimal operating conditions for the system and to identify a region that contains certain operational parameters desired by the researcher. For these reasons, it is possible to apply this method to determine the optimum values of each factor that provide the best biosorption capacity for each sorbent for each metal (Table 6).

**Table 6:** Optimum values for each factor that maximize the response variable (*q*)

**Biosorbent**	**Factor**			
	**Co(II)**		**Ni(II)**	
	**[Co(II)] (mg L**^**−1**^**)**	***X***_**0**_** (g)**	**[Ni(II)] (mg L**^**−1**^**)**	***X***_**0**_** (mg)**
Strain C-1	39.1	0.0061	39.3	1
Strain 16	35.9	0.0064	49.9	7.2
Strain 19	39.8	0.0066	20.6	1

The optimum values of initial biomass and metal concentration that maximized the Co(II) biosorption proved to be very similar for all three biosorbents studied, as would be expected considering the similarities in the corresponding contour plots. In all cases, a maximum Co(II) uptake was obtained with mid-range values for initial biomass and for initial concentration values below the maximum of 50 mg L^−1^ within the defined range. This situation reflects the fact that the biosorbents behave according to the Langmuir model, which means that saturation takes place at high metal concentrations. On the other hand, different values were obtained for the three biosorbents for the optimization of Ni(II) uptake. For biosorbents from strains C-1 and 19, the optimum initial biomass values were the lowest, whereas the optimum initial concentrations of Ni(II) required to maximize the biosorption capacity for this metal were mid-range values. This difference could be related to the fact that, when a few particles of the biosorbent are present, there should be a larger number of active sites available for interaction with the sorbate. Conversely, for *S. marcescens* strain 16 biomass, the optimum Ni(II) concentration is almost the highest value within the studied range, while the optimum initial biomass values are mid-range, i.e., higher metal concentrations are required in order to achieve the best biosorption performance.

## Conclusions

A study of the Ni(II) and Co(II) biosorption capacity of native metal–resistant *S. marcescens* strains C-1, 16, and 19 showed recovery values comparable to, and in some cases higher than, those reported for other *Enterobacteriaceae*. The sorption kinetics in monometallic systems was fast, and equilibrium was reached within 2 h of contact. The experimental data fitted best to the Langmuir mathematical model, and the parameters *q*_max_ and *b* showed that the three biosorbents had a higher affinity for Co(II) ions. The maximum amount of metal adsorbed was obtained for *S. marcescens* strain 19 biomass, with recoveries of 21.33 mg g^−1^ for Co(II) and 15.98 mg g^−1^ for Ni(II) ions. The use of an experimental design with the response surface methodology allowed the analysis of factors that influence the biosorption performance, as well as their interactions, and this approach was used to predict the optimum values to maximize metal uptake. However, further studies are required in order to increase the values obtained for the regression coefficient so that the model can be validated for these biosorbents. These results are significant since very little research concerning *Serratia* spp. metal biosorption has been reported in the literature, especially for Ni(II) and Co(II) ions. This work also contributes to our understanding of the potential uses of serpentine microbial populations for the design of environmental cleanup technologies.

## Data Availability

All data generated or analyzed during this study are included in this published article.

## Declarations

### Novelty statement

Biosorption represents a feasible alternative for an eco-friendly and cost-effective technology that could help to minimize the harmful environmental impact generated by liquid residues from the industrial activities. Although biosorption has been extensively studied for many years, the process is still only carried out on the laboratory scale. For this reason, the study of biosorption is a high priority in order to optimize the process. This paper aimed to optimize nickel and cobalt biosorption from aqueous solutions using three native metal–resistant *Serratia marcescens* strains. Initial pH, metal concentration, biomass, and temperature were optimized by Plackett–Burman method, and, finally, biomass and metal concentration were studied using response surface methodology (RSM) to improve nickel and cobalt biosorption. There are only few works about the use of *Serratia marcescens* as biosorbent.
